# Avoidant attachment attenuates the need-threat for social exclusion but induces the threat for over-inclusion

**DOI:** 10.3389/fpsyg.2022.881863

**Published:** 2022-08-16

**Authors:** Tsubasa Izaki, Wei Wang, Taishi Kawamoto

**Affiliations:** ^1^Human Informatics and Interaction Research Institute, National Institute of Advanced Industrial Science and Technology, Tsukuba, Japan; ^2^Graduate School of Integrated Arts and Sciences, Hiroshima University, Hiroshima, Japan; ^3^Institute of Psychology and Behavior, Faculty of Education, Henan University, Kaifeng, China; ^4^College of Humanities, Chubu University, Kasugai, Japan

**Keywords:** avoidant attachment, social exclusion, over-inclusion, fundamental needs, attachment style

## Abstract

The influence of attachment style—anxious (AX) and avoidant (AV) attachment—on subjective responses to socially excluded experiences termed “Need-Threat” remains inconsistent. Need-Threat is a composite score of four fundamental needs: belonging, self-esteem, control, and meaningful existence. Individuals with high AX tend to spend much effort maintaining strong connections with others, while those with high AV tend to maintain high levels of self-esteem by distancing themselves from others. Therefore, attachment style is most likely to influence the need associated with each style. In addition, since individuals with high AV satisfy their needs by keeping independence from others, they would experience the Need-Threat against excessive inclusion from others. This study aimed to investigate the influence of attachment style on each Need-Threat response to various inclusionary statuses. A total of 133 undergraduate students were equally assigned to low or high groups for each attachment style. Participants played one of the three types of the cyberball task (a ball-tossing game with programmed players): excluded, included, or over-included situation. The high AV group felt fewer threats to self-esteem than the low AV group in the excluded situation (*p* = 0.02). Furthermore, only in the over-included situation did the high AV group feel more threats to belonging and self-esteem than the low AV group (*ps* < 0.02). AX did not influence any situation. These findings suggest that individuals with high AV would have a restrictive alleviation effect on adverse subjective responses to socially excluded experiences but demonstrate maladaptive subjective responses to over-included experiences.

## Introduction

Attachment style is a personal attitude toward others and a means of building relationships with others (Bartholomew and Horowitz, 1991; Brennan et al., 1998). The purpose of the attachment system is to provide safety by regulating proximity-seeking behavior, which allows for the attainment of care and support from an attachment figure (Bowlby, 1969/1982). People whose needs for proximity are satisfied to experience attachment securely and tend to expect these responses from other relationship partners. However, when needs for proximity are discouraged or go unmet, people experience attachment insecurity, and the insecure attachment style (anxious or avoidant attachment) is formed to attempt other coping strategies (Griffin and Bartholomew, 1994; Brennan et al., 1998). Anxious attachment (AX) assesses individuals’ beliefs about self-worth and whether they will be included by others, while avoidant attachment (AV) assesses individuals’ beliefs about others and whether they feel comfortable approaching them (Mikulincer and Shaver, 2016). Some people display higher levels of each style, while others are low in both styles of attachment (i.e., secure attachment style).

According to the optimal calibration hypothesis (Chester et al., 2012), early life history shifts one’s neural responses to social exclusion. Social exclusion is an experience that attenuates relationships with others; someone is ostracized, rejected, and ignored by belonging groups or acquaintances (Macdonald and Leary, 2005). The optimal calibration hypothesis argues that individuals with high AX experienced unpredictable social rejection by parents in their early life history, which caused a “hyper-activated” response. High-AX individuals take the hyper-activation strategies, which are “fight” responses to frustrated attachment needs. They do not easily give up on proximity-seeking but rather intensify it to demand or force the attachment figure’s attention, love, and support (Mikulincer and Shaver, 2016). In contrast, individuals with high AV experienced chronic social rejection by parents in their early life, which resulted in a “deactivated” response (Chester et al., 2012). High AV individuals take the deactivation strategies, which are a “flight” reaction to an attachment figure’s unavailability. Their attachment system is downregulated to avoid frustration and distress caused by the attachment figure’s unavailability (Mikulincer and Shaver, 2016). The previous study found that high-AX individuals showed heightened activities in the dorsal anterior cingulate cortex (dACC) and anterior insula during excluded experiences, whereas high-AV individuals showed dampened activities in these regions (DeWall et al., 2012). Activities of the dACC and anterior insula positively correlated with the subjective negative responses to excluded experiences (e.g., Eisenberger et al., 2003; Onoda et al., 2010). This suggests that the dACC and anterior insula alarm the socially excluded in situations using unpleasantness (neural alarm system; Eisenberger and Lieberman, 2004). Since attachment style modulates activity in the dACC and anterior insula (DeWall et al., 2012), it also affects subjective responses to social exclusion.

Yaakobi and Williams (2016a,b) found that during excluded experiences, participants with high AX felt more distressed, and those with high AV felt less distressed compared to those with low AX and AV, respectively. In addition, AX moderated the mediation of death anxiety on social exclusion-evoked distress (Yaakobi, 2019), and high-AX participants had more loss of meaning in life after excluded experiences (Shaver and Mikulincer, 2013). These findings are in line with the characteristics of the respective styles and suggest that AX tends to enhance subjective responses against social exclusion, whereas AV tends to attenuate the responses. On the other hand, the study showing the effect of attachment style on the neural activity to social exclusion found no influence on subjective responses (DeWall et al., 2012). Similar results were found in other studies (McDonald and Donnellan, 2012; Izaki and Ogawa, 2017; Izaki et al., 2017). Taken together, the influence of attachment style on subjective responses to social exclusion remains inconsistent for both styles.

Previous studies on attachment style and social exclusion measured the negative subjective responses using the Need-Threat scale (Williams et al., 2000; Williams, 2009). The Need-Threat scale assesses the threat to four fundamental needs: belonging, self-esteem, control, and meaningful existence. Belonging is the desire for frequent, positive, and stable interactions with others (Baumeister and Leary, 1995). Self-esteem is the belief that oneself is good and worthy (Steele, 1988). Control is the desire to control one’s interactions with others (Peterson and Seligman, 1984). Meaningful existence is the desire to maintain the meaning of one’s existence, which can be confirmed through receiving attention or recognition from others (Greenberg et al., 1986). Previous studies examined the effect of attachment style on a composite Need-Threat score of four needs (DeWall et al., 2012; McDonald and Donnellan, 2012; Yaakobi and Williams, 2016a,b; Izaki and Ogawa, 2017; Izaki et al., 2017). However, it would be suitable to investigate the effect on each need score rather than a composite score since the attachment style has a related need, respectively. High-AX individuals tend to spend more effort on maintaining strong connections with others than low-AX individuals (Mikulincer and Shaver, 2003). Thus, high-AX individuals may easily feel a threat to belonging *via* excluded experiences. In contrast, high-AV individuals tend to maintain high levels of self-esteem by distancing themselves from others compared to low-AV individuals (Fraley et al., 1998). Thus, it may be unlikely for high-AV individuals to feel a threat to self-esteem, even if they are excluded. This study aimed to investigate the effect of attachment style on the need associated with each style to isolate the specific need influenced by attachment style.

Regarding the AV response, high-AV individuals would feel a threat to fundamental needs by being included by others because they tend to explicitly reject or minimize the importance of emotional attachments, passively avoid close relationships, and strive for self-reliance and independence (Bartholomew, 1990; Griffin and Bartholomew, 1994; Collins and Feeney, 2000). Previous studies found that high-AV participants felt more Need-Threat against inclusion from others than low-AV participants (Yaakobi and Williams, 2016a,b). In contrast, our previous studies found no influence of AV on the Need-Threat to socially included experiences (Izaki and Ogawa, 2017; Izaki et al., 2017). In these studies, participants played the cyber ball task (Williams et al., 2000; Williams and Jarvis, 2006), which reproduces socially excluded or included situations. Participants played a ball-tossing game with two or more computer-generated players. In the socially included situation of the task, a participant receives a ball at the same frequency as other players, and thus, he or she may find it difficult to feel included by others. This ambiguity may have caused an inconsistency between the studies. This study set the over-inclusion situation in addition to the equal inclusion situation to investigate the effect of AV on the Need-Threat against situations that can be recognized as included and whether the effect of AV differs depending on the degree of inclusionary status (i.e., equal vs. over-inclusion).

This study aimed to (1) examine the effect of attachment style on a threat to each fundamental need for excluding experiences and (2) examine the effect of AV on the Need-Threat after over-including experiences. To achieve these aims, this study used the cyberball task. We hypothesized that: (1) after socially excluded cyberball tasks, high-AX individuals would feel a greater threat to belonging than low AX ones. (2) After socially excluded cyber ball tasks, high-AV individuals would feel less threat to self-esteem than low-AV individuals. (3) After socially over-included cyberball tasks, high-AV individuals would feel more threat to their needs. This study would promote further understanding of the distress experienced by individuals with insecure attachment against positive and negative inclusionary statuses.

## Methods

### Attachment style score

Each attachment style was assessed using the Experiences in Close Relationship Questionnaire for the Generalized Other (ECR-GO; Nakao and Kato, 2004). ECR-GO assessed the anxious (e.g., “I worry that others won’t care about me as much as I care about them”) and avoidant (e.g., “I prefer to depend on myself rather than other people”) dimensions of attachment. The ECR-GO consists of 30 items, each answered using a seven-point Likert scale (ranging from 1 = “totally disagree” to 7 = “totally agree”). Items were reverse-coded when appropriate and averaged to create a composite score, with a higher score representing higher anxious or avoidant attachment. While people often show greater levels of one attachment style, it is also common for anxious and avoidant attachments to overlap (Fraley, 2012; Beck and Clark, 2009). Thus, it is possible to investigate the effects of each type of attachment style on the same individual (DeWall et al., 2012).

### Participants and screening

In total, 153 undergraduate students (98 females and 55 males, *M*_*age*_ = 20.40 ± 1.80) applied to participate in this study. The students completed the ECR-GO (Cronbach’s α = 0.89 for AX, α = 0.86 for AV). For each attachment style, they were stratified into three percentile groups (0–33 percentile; 33–66 percentile; 66–100 percentile; 51 students each) based on their attachment style scores. These groups were designated as the low (*M*_*anxiety*_ = 2.57 ± 0.44, *M*_*avoidance*_ = 2.70 ± 0.38), middle (*M*_*anxiety*_ = 3.60 ± 0.24, *M*_*avoidance*_ = 3.75 ± 0.29), and high groups (*M*_*anxiety*_ = 4.58 ± 0.51, *M*_*avoidance*_ = 4.93 ± 0.61), and those in the low and high groups participated in this experiment. Since 71 students met the thresholds for both attachment styles, and 62 students met the thresholds of either attachment style, the final number of participants in this experiment was 133 (87 females and 46 males, *M*_*age*_ = 20.46 ± 1.81). The participants were informed about the experiment in advance; they provided their written consent to participate therein. The Research Ethics Committee of the Graduate School of Integrated Arts and Sciences of Hiroshima University, Japan (25-5) approved the study protocol.

### Self-reported assessments

To assess a threat to four fundamental needs, participants completed the Japanese version of the Need-Threat scale (Izaki et al., 2017), consisting of 16 items covering all four needs: belonging (e.g., “I felt rejected”), self-esteem (e.g., “I felt good about myself”), control (e.g., “I felt I had control throughout the game”), and meaningful existence (e.g., “I felt meaningless”). Items were reverse-coded when appropriate and summed to create a threat score for each fundamental need (Cronbach’s α = 0.88 for belonging, α = 0.73 for self-esteem, α = 0.62 for control, α = 0.83 for meaningful existence). Higher scores indicate a greater level of threat. Moreover, to investigate the influence of attachment style on participants’ cognitive performance, the perception of their inclusionary status was assessed by an open question (“Assume that 33% of the time you would receive the ball if everyone received it equally, what percent of the throws did you receive?”).

### Cyberball task and protocols

To reproduce various inclusionary situations, we used the cyberball task (Williams et al., 2000; Williams and Jarvis, 2006). In this task, participants played a ball-tossing game with two computer-generated players. One task consisted of 30 throws and lasted approximately 5 min. Participants were randomly assigned to one of three situations: (1) Excluded situation: Participants received the ball once from each of the two other players at the beginning (i.e., two throws) and then never again. (2) Included situation: Participants received one-third of the total (i.e., 10 throws). (3) Over-included situation: Participants received twice as many balls as the other players (i.e., 15 throws). After entering the laboratory, participants were told that they would play an online game with two other undergraduate participants. Thereafter, they played the cyberball task in the assigned situation. After the task, participants answered the Need-Threat scale and the open question. At the end of the experiments, we debriefed participants, explaining to them that other players were computer-generated. We also disclosed to them the true purpose of the experiment. The total duration of the experiment was approximately 15 min.

### Statistical analysis

All statistical analyses were computed using SPSS (version 22.0; IBM, Unite States). To confirm the validity of grouping and no difference in each attachment score between situations and groups, a one-way analysis of variance (ANOVA) with the *situation* (excluded vs. included vs. over-included situations) and *group* (low vs. high groups) as the main factors were performed on each attachment style.

Since the correlation between anxious attachment and avoidant attachment scores was marginally significant (*r* = 0.146, *p* = 0.09), a two-way analysis of covariance (ANCOVA) with other style scores as a covariant was performed on all dependent variables. To investigate the effect of each attachment style on the four fundamental needs, ANCOVA with the *situation* (excluded vs. included vs. over-included situations) and *group* (low vs. high groups) as the main factors was performed for each attachment style, controlling for other style scores. A similar analysis was performed on the perceived percentage of throws to check whether there is a difference in the perception of a number of balls received between the high and low groups of AX or AV. When a significant effect was confirmed by the ANCOVA, multiple comparisons based on Holm’s method were performed. A *p*-value of <0.05 was considered statistically significant.

## Results

### Anxious attachment

For anxious attachment scores, a situation × AX group ANOVA revealed the main effect of group [*F*_(1, 96)_ = 427.18, η_*p*_^2^s = 0.82, *p* < 0.001] but not the main effect of the situation [*F*_(2, 96)_ = 0.38, *p* = 0.68] and the interaction effect between the situation and group [*F*_(2, 96)_ = 0.07, *p* = 0.93]. These indicate that the high- and low-AX groups were adequately divided, and the AX scores were similar among three situations.

### Threat to four fundamental needs

**FIGURE 1:**
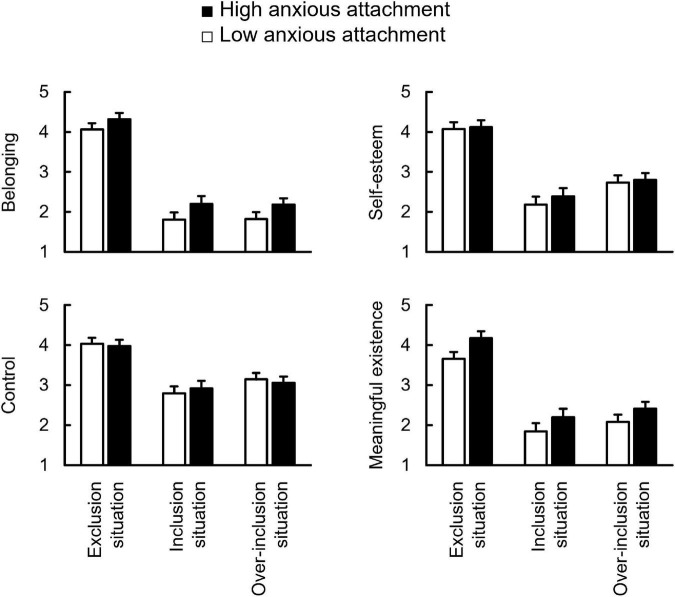
Comparison between low and high anxious attachment of need-threat score for belonging, self-esteem, control, and meaningful existence in three situations. Error bars indicate the standard error of means across participants.

Figure 1 summarizes the effects of AX on threats to the four fundamental needs for excluded, included, and over-included situations of the cyberball task. A situation × AX group ANCOVA (controlling avoidant attachment score) revealed the main effects of situation on all needs [*Fs*_(2, 95)_ > 28.48, *ps* < 0.001, η_*p*_^2^s > 0.38]. The *post hoc* test indicated that participants in the excluded situation felt more threat to all needs than those in other situations [*ts*(95) > 5.86, *ps* < 0.001, *ds* > 1.36]. Furthermore, those in included and over-included situations experienced similar threats to all the needs (*ts* < 1.40, *ps* > 0.17). The ANCOVA revealed the main effects of the AX group on belonging and meaningful existence [*Fs*_(1, 95)_ > 6.84, *ps* < 0.01, η_*p*_^2^s > 0.07] but not on self-esteem and control (*Fs* < 0.69, *ps* > 0.41). This indicates that the high-AX group felt higher threats from these needs than the low AX group, regardless of the situation. There was no interaction effect on all needs (*Fs* < 0.25, *ps* > 0.78).

### Perceived percentage of throws

A situation × AX group ANCOVA (controlling avoidant attachment score) for perceived percentages of throws revealed a main effect of situation [*F*_(2, 95)_ = 113.14, *p* < 0.001, η_*p*_^2^ = 0.70]. The *post hoc* test indicated that participants in excluded situation estimated a lower perceived percentage of throws than those in other situations [*ts*(95) > 12.55, *ps* < 0.001, *ds* > 3.03], and no difference was evident between those in included and over-included situations (*t* = 0.36, *p* = 0.72). Furthermore, there was no main effect of AX group and no interaction effect (*F*s < 0.48, *ps* > 0.62).

### Avoidant attachment

For avoidant attachment scores, a situation × AV group ANOVA revealed the main effect of group [*F*_(1, 96)_ = 473.30, *p* < 0.001, η_*p*_^2^s = 0.83] but not the main effect of the situation [*F*_(2, 96)_ = 0.57, *p* = 0.57] and the interaction effect between the situation and group [*F*_(2, 96)_ = 0.11, *p* = 0.90]. These indicate that the high and low AV groups were adequately divided, and the AV scores were similar among three situations.

### Threat to four fundamental needs

**FIGURE 2:**
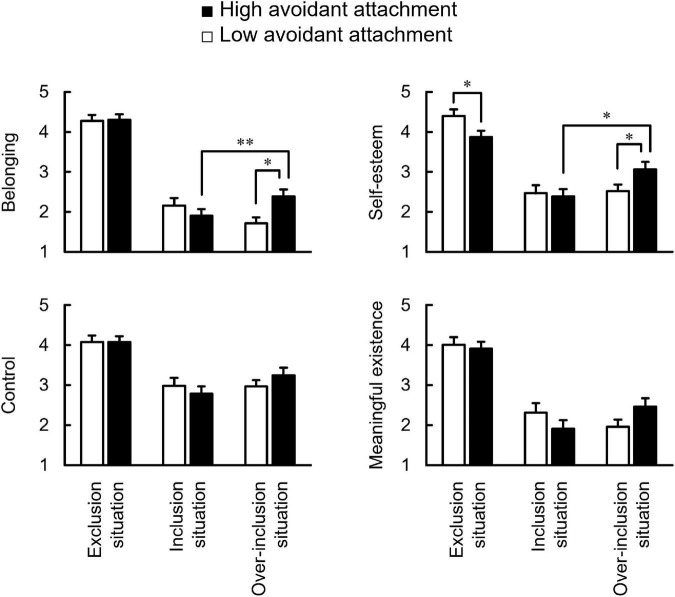
Comparison between the low and high avoidant attachment of need-threat score for belonging, self-esteem, control, and meaningful existence in three situations. Error bars indicate the standard error of means across participants. *p < 0.05, ^**^p < 0.01.

Figure 2 summarizes the effects of AV on threats to the four fundamental needs for excluded, included, and over-included situations of the cyberball task. A situation × AV group ANCOVA (controlling anxious attachment score) revealed the main effects of situation on all needs [*Fs*_(2, 95)_ > 29.50, *ps* < 0.001, η_*p*_^2^s > 0.38]. The *post hoc* test indicated that participants in the excluded situation felt more threats to all needs than those in included and over-included situations [*ts*(95) > 5.99, *ps* < 0.001, *ds* > 1.39]; however, those in included and over-included situations experienced similar threats to all needs (*ts* < 1.96, *ps* > 0.06). As expected, the ANCOVA revealed significant interaction effects between threats to belonging and self-esteem [*Fs*_(2, 95)_ > 4.17, *ps* < 0.02, η_*p*_^2^s > 0.08]. The *post hoc* test revealed the simple main effects of over-inclusion situation on both needs [*Fs*_(1, 95)_ > 5.17, *p* < 0.02, η_*p*_^2^ > 0.15], indicating that the high-AV group felt more threats to belonging and self-esteem than the low-AV group. Simple main effects on the high-AV group were also revealed in both needs [*Fs*_(2, 95)_ > 17.40, *ps* < 0.001, η_*p*_^2^s > 0.43], indicating that the high-AV participants in over-included situation felt more threats to belonging and self-esteem than those in included situation [*ts*(95) > 2.13, *ps* < 0.04, *ds* > 0.05]. Notably, a simple main effect of excluded situation was revealed only for threats to self-esteem [*F*_(1, 95)_ = 5.50, *p* = 0.02, η_*p*_^2^ = 0.13], indicating that the high-AV group felt fewer threats to self-esteem during excluded situation than the low AV group. There was no main effect of the AV group for all needs (*Fs* < 1.27, *ps* > 0.26).

### Perceived percentage of throws

A situation × AV group ANCOVA (controlling for the anxious attachment score) for perceived percentages of throws revealed a main effect of situation [*F*_(2, 95)_ = 50.58, *p* < 0.001, η_*p*_^2^ = 0.52]. The *post hoc* test indicated that participants in excluded situation estimated lower perceived percentages of throws than those in other situations [*ts*(95) > 8.44, *ps* < 0.001, *ds* > 1.99]. There was no difference in the perceived percentage of throws between included and over-included situations (*t* = 0.42, *p* = 0.68). A main effect of AV group and an interaction effect were not revealed (*Fs* < 0.58, *ps* > 0.56).

## Discussion

Thus far, the influence of attachment style—anxious and avoidant attachment—on the Need-Threat evoked by various degrees of inclusionary situations remains inconsistent. This study investigated how attachment style influences the need associated with each style, and how high-AV individuals feel a threat to their needs when over-included. To address these issues, in this study, a threat to each fundamental need was evoked by the cyberball task, and the over-included situation was added to excluded and included situations. The major findings were as follows: (1) the high AV group felt fewer threats to self-esteem than the low AV group in the excluded situation and (2) The high AV group felt more threats to belonging and self-esteem than the low AV group in the over-included situation, but not in the included situation. Collectively, our findings suggest that high AV individuals would have a restrictive attenuative effect on adverse subjective responses to excluded experiences but show maladaptive subjective responses to over-included experiences.

### Avoidant attachment

As postulated in the hypothesis, this study showed the effect of AV on only threats to self-esteem against social exclusion (Figure 2). This finding replicates and extends the results of the previous studies showing the effect of AV on the composite Need-Threat (Yaakobi and Williams, 2016a,b). As high AV individuals are not convinced of the availability of emotional support from others, they maintain a high level of self-esteem by striving for independence and emotional distance from others (Fraley et al., 1998; Mikulincer and Shaver, 2016). According to research on the brain, greater activity in brain regions involved in social exclusion (dACC, anterior insula) was associated with lower self-esteem (Eisenberger et al., 2011). Furthermore, activities in these regions for excluded situations had negative correlations with avoidant attachment (DeWall et al., 2012). Combining these previous studies with this study suggests that high AV individuals suppress a threat to self-esteem by attenuating an activity of exclusion-related brain regions during excluded experiences. The four fundamental needs can be classified into needs (belonging, control, and meaningful existence) related to relationships with others and needs (self-esteem) related to a belief in oneself (Williams and Sommer, 1997). The current findings indicate that although high-AV individuals adopt deactivation strategies of the attachment system, only a threat to the need related to the belief in oneself (i.e., self-esteem) may be suppressed; other needs related to relationships with others may be threatened. This aligns with previous studies that suggest that high-AV individuals possess the need to belong, similar to people with secure attachment (Carvallo and Gabriel, 2006) and suggest that it is necessary to ameliorate from social exclusion by improving belonging status through socially included experiences, even in high-AV individuals (Izaki et al., 2017).

When they were over-included, the high AV participants felt more threats to belonging and self-esteem than in other situations. The low-AV participants felt more threats in the over-included situation (Figure 2). These findings support the hypothesis that they feel a threat to be included excessively. As described above, high AV individuals tend to keep away from others to maintain their self-esteem (Main and Weston, 1982). Therefore, because high AV participants paid attention to positive characteristics, intentions, and related behavior of other players by receiving twice as many balls as others, they became conscious of their suppressed negative aspects. Consequently, their self-esteem might have been threatened. Moreover, since self-esteem functions as a sociometer of social inclusion (Leary et al., 1995), the inclusionary status was unintentionally underestimated by the feeling of the threats to self-esteem, and, thus, their belonging might also have been threatened. On the other hand, the influence of AV was not observed in the included situation unlike the previous studies (Yaakobi and Williams, 2016a,b), indicating that AV would not have a robust influence over the unobtrusive inclusion. High-AV individuals possess the need to belong because positive feelings and self-esteem increased compared to the baseline by noticing they were included by others (Carvallo and Gabriel, 2006). Therefore, high-AV individuals are unlikely to feel a threat when included, to the same extent as low-AV individuals. Previous studies showed that high-AV individuals shift their attention away from stimuli depicting or evoking attachment-related themes (e.g., pictures of one’s mother), suggesting that they tend to look away from attachment-related stimuli to avoid arousing painful memories (Main and Solomon, 1990; Kirsh and Cassidy, 1997). The current findings suggest that although high-AV individuals usually maintain a normal mind by distracting attention from others, they cannot help feeling a threat when their attention is unintentionally allocated to others, by experiencing inclusion from others beyond necessity.

### Anxious attachment

Contrary to the hypothesis, anxious attachment did not affect any fundamental needs (Figure 1). In the cyberball task, because the Need-Threat is easily evaluated to be higher regardless of an individual’s characteristics, it may be difficult to show the influence of individual differences on subjective responses. Indeed, many studies have not shown the influence of various individual differences, including AX on subjective responses during cyberball tasks (e.g., attachment style, DeWall et al., 2012; depression, Kumar et al., 2017; self-esteem, McDonald and Donnellan, 2012; rejection sensitivity, Beekman et al., 2016). However, several studies have shown that AX influences neural responses to social exclusion. For example, high-AX individuals showed increased activities in dACC and anterior insula during the cyberball task (DeWall et al., 2012) and increased left amygdala activity in response to the rejective feedback expressed by an angry face on one’s performance (Vrtička et al., 2008). Collectively, the effects of individual differences (e.g., AX) that enhance the Need-Threat to social exclusion would be difficult to show in the cyberball task, unlike the effects of individual differences (e.g., AV) that attenuate the Need-Threat.

In addition, AX did not influence the Need-Threat for social inclusion and over-inclusion (Figure 1). This finding is aligned with that of our study showing that AX did not affect the Need-Threat and the event-related brain potential during included experiences (Izaki et al., 2017). Taken together, just because high-AX individuals tend to be hypervigilant for the signs of rejection or abandonment (e.g., Spielmann et al., 2013; Mikulincer and Shaver, 2016), it does not mean that they feel extremely comfortable being included by others regardless of the degree of inclusion.

### Impact of the individual differences in responses to social inclusion

Individuals with high AV felt threats to belonging and self-esteem when they were overly included (Figure 2). On the other hand, individuals with borderline personality disorder (BPD) felt discomforted during the cyberball task more than healthy individuals in the excluded and included situations, but not in the over-included situation (De Panfilis et al., 2015), suggesting that their heightened socially painful feelings will decrease only when their interaction partners demonstrate an over-including attitude. The findings of the present and previous studies indicate that individual differences affect responses to over-inclusion as with exclusion. An event that needs to consider the effect of individual differences on over-inclusion is the amelioration from exclusion through included experiences. The adverse effects of excluded experiences (e.g., Need-Threat) are subsequently attenuated by being included in social situations (Tang and Richardson, 2013; Izaki et al., 2017). However, this method would not be suitable for all excluded individuals. High-AV individuals may need to be included moderately, while those with BPD may need to be included excessively. We propose that it is important to consider the frequency of inclusion based on individual characteristics to develop an ameliorative approach for socially excluded individuals.

### Limitations and future directions

Some limitations are involved in this study. First, statistical analysis for the dataset of this study using a linear model may be more reasonable because the attachment style score is a continuous variable. However, the dataset was not suitable for analysis by a linear model because this study had no data of individuals with attachment scores near the mean (i.e., middle 33–66 percentile group). Second, our design was a between-subject design. Since this study has no baseline data, we cannot rule out that the characteristics of the participants may have been biased between the situations. Although there was no difference in attachment scores between the situations, future studies should verify this possibility using a within-subject design. Third, in the present and previous studies (Yaakobi and Williams, 2016a,b), the effect of avoidant attachment on responses to included experiences was shown only by subjective measures (i.e., the Need-Threat scale). It should be examined whether avoidant attachment also modulates neural responses during included or over-included experiences and/or alters subsequent behaviors (e.g., prosocial behavior, aggression). Finally, this study did not consider the effects of other psychological factors and personality variables that might influence response to social situations (e.g., self-esteem; Onoda et al., 2010), and this should be explained in a future study.

This study demonstrated the negative impact of attachment styles, specifically AV, on responses to socially over-included experiences. Future studies should conduct additional experiments to anchor the impact, to achieve a more comprehensive understanding of the phenomenon. Mikulincer and Shaver (2001) found that priming a secure base reduced participants’ negative reactions to outgroups without regard to their attachment styles. In a related line of research, Karremans et al. (2011) examined whether reminders of secure attachment relationships could attenuate neurophysiological pain- and stress-related responses to social exclusion reproduced by the cyberball task. As a result, less activation in brain areas implicated in the regulation and experience of social distress (e.g., lateral and medial prefrontal cortex) was found to the extent that individuals felt more securely attached to their attachment figure. If individuals with high AV feel distressed upon being over-included as shown in this study (Figure 2), excessive secure base priming would not bring the attenuative effect of social distress against social exclusion, and, on the contrary, is likely to enhance their distress.

## Conclusion

In this study, high-AV individuals felt fewer threats to self-esteem after excluded experiences, whereas they felt threats to belonging and self-esteem after over-included experiences. These findings suggest that avoidant attachment has a restrictive attenuative effect on adverse subjective responses to excluded experiences but has a maladaptive effect on subjective responses to excessive included experiences. This study encourages us to improve our understanding of social inclusion and to consider the influence of individual differences to make good use of the benefits of social inclusion.

## Data availability statement

The raw data supporting the conclusions of this article will be made available by the authors, without undue reservation.

## Ethics statement

The studies involving human participants were reviewed and approved by the Research Ethics Committee of the Graduate School of Integrated Arts and Sciences, Hiroshima University. The patients/participants provided their written informed consent to participate in this study.

## Author contributions

TI and TK conceived and designed the research, performed the experiments, and interpreted the results of experiments. TI analyzed the data, prepared the figures, and drafted the manuscript. TI, WW, and TK edited and revised the manuscript and approved the final version of the manuscript. All authors contributed to the article and approved the submitted version.
